# Sorafenib Metabolism Is Significantly Altered in the Liver Tumor Tissue of Hepatocellular Carcinoma Patient

**DOI:** 10.1371/journal.pone.0096664

**Published:** 2014-05-05

**Authors:** Ling Ye, Xiaoshan Yang, Enshuang Guo, Weiying Chen, Linlin Lu, Ying Wang, Xiaojuan Peng, Tongmeng Yan, Fuyan Zhou, Zhongqiu Liu

**Affiliations:** 1 Department of Pharmaceutics, School of Pharmaceutical Sciences, Southern Medical University, Guangzhou, Guangdong, China; 2 International Institute for Translational Chinese Medicine, Guangzhou University of Chinese Medicine, Guangzhou, Guangdong, China; 3 Department of Infectious Diseases, Nanfang Hospital, Southern Medical University, Guangzhou, Guangdong, China; Macau University of Science and Technology, Macau

## Abstract

**Background:**

Sorafenib, the drug used as first line treatment for hepatocellular carcinoma (HCC), is metabolized by cytochrome P450 (CYP) 3A4-mediated oxidation and uridine diphosphate glucuronosyl transferase (UGT) 1A9-mediated glucuronidation. Liver diseases are associated with reduced CYP and UGT activities, which can considerably affect drug metabolism, leading to drug toxicity. Thus, understanding the metabolism of therapeutic compounds in patients with liver diseases is necessary. However, the metabolism characteristic of sorafenib has not been systematically determined in HCC patients.

**Methods:**

Sorafenib metabolism was tested in the pooled and individual tumor hepatic microsomes (THLMs) and adjacent normal hepatic microsomes (NHLMs) of HCC patients (n = 18). Commercial hepatic microsomes (CHLMs) were used as a control. In addition, CYP3A4 and UGT1A9 protein expression in different tissues were measured by Western blotting.

**Results:**

The mean rates of oxidation and glucuronidation of sorafenib were significantly decreased in the pooled THLMs compared with those in NHLMs and CHLMs. The maximal velocity (*V_max_*) of sorafenib oxidation and glucuronidation were approximately 25-fold and 2-fold decreased in the pooled THLMs, respectively, with unchanged *K_m_* values. The oxidation of sorafenib in individual THLMs sample was significantly decreased (ranging from 7 to 67-fold) than that in corresponding NHLMs sample. The reduction of glucuronidation in THLMs was observed in 15 out of 18 patients’ samples. Additionally, the level of CYP3A4 and UGT1A9 expression were both notably decreased in the pooled THLMs.

**Conclusions:**

Sorafenib metabolism was remarkably decreased in THLMs. This result was associated with the down regulation of the protein expression of CYP3A4 and UGT1A9.

## Introduction

Hepatocellular carcinoma (HCC) is one of the most common hepatic malignancies in regions where chronic hepatitis or liver diseases are prevalent, such as in China [1], [2]. Sorafenib, an orally active multikinase inhibitor, has been approved in the United States and the European Union for the treatment of HCC [2]. Sorafenib enhances the overall survival compared with placebo in advanced HCC patients [3]. Like other anti-tumor medications, sorafenib is associated with several of side effects, including diarrhea, nausea, fatigue, hypertension and dermatologic toxicities. And hand-foot skin reaction (HFSR) is currently emerging as a major toxicity of sorefnib treatment, with the greatest frequency and the greatest morbidity [4]–[6].

Sorafenib metabolism occurs primarily in the liver by cytochrome P450 (CYP) 3A4-mediated oxidation and uridine diphosphate glucuronosyl transferase (UGT) 1A9-mediated glucuronidation [2]. Sorafenib has a mean elimination half-life ranging from approximately 25 h to 48 h. Approximately 77% of the administrated sorafenib dose is detected in feces (50% as unchanged drug) and 19% is excreted in urine, almost exclusively as glucuronide conjugates of the parent drug or metabolites, but not unchanged sorafenib [7]. Sorafenib N-oxide (M2) accounts for ∼17% of the circulating analytes in the plasma and is the major CYP 3A4 metabolite. It has potency similar to that of sorafenib. N-oxidation plus N-methylhydroxylation (M1), N-methylhydroxylation (M3), N-demethylation (M4), and N-oxidation plus N-demethylation (M5) have also been detected as CYP3A4 metabolites of sorafenib. In addition, M7 accounts for ∼15% of the administrated dose of sorafenib and is glucuronidated by UGT1A9 [8]–[10]. A previous study has reported that plasma sorafenib concentration is increased by inhibiting CYP3A4 in combination with felodipine in a patient with HCC [11]. Moreover, prednisolone, a CYP3A4 inducer, can stimulate sorafenib metabolism [12]. Therefore, changes in sorafenib metabolism by altering CYP3A4 or UTG1A9 activity may affect its clinical effects and sorafenib-induced toxicity.

The phases I (CYPs) and II (UGTs) metabolizing enzymes have vital roles in carcinogenesis and tumor response to anticancer therapy [13]–[15]. CYPs and UGTs are present in many organs and tissues, but their concentration is most abundant in the liver. Hepatic CYPs and UGTs are involved in the pathogenesis of several liver diseases [1]. And drug metabolism mediated by CYPs and UGTs is impaired in patients with liver disease [16].

Liver diseases are associated with reduced CYP and UGT activities. Hepatic steatosis has been associated with decreased hepatic CYP3A activity in humans [8]. CYP3A4 and UGT activities declined in patients with hepatic cirrhosis [15], [17]. Consequently, the ability of the liver to eliminate many clinical therapeutic drug substrates would decline. Among patients with liver cirrhosis, several pharmacokinetics studies have shown a significant decrease in the metabolism of drugs (lidocaine, nifedipine and midazolam etc.) metabolized by CYP3A4 [18]–[20]. Atomoxetine, a drug primarily eliminated via CYP2D6, was with reduced metabolism in patients with hepatic impairment [21]. And the metabolic clearances of omeprazole, s-mephenytoin and aminopyrine metabolized by CYP2C19, have been reported to be decreased in patients with cirrhosis [22]–[25]. The clearance of CYP2A6 substrates was decreased for hepatitis A infections [26]. The metabolic clearance of zidovudine, a drug glucuronidated by UGT2B7, was decreased significantly in liver cirrhosis (0.17 versus 0.37 ml/min/mg protein) [17]. The in vivo oral clearance of zomepirac undergoing extensive glucuronidation, was decreased by 50% in cirrhosis [27]. These findings have practical implications for the use of drugs in patients with liver diseases and emphasize the need to understand the metabolism of therapeutic compounds.

However, the metabolism characteristic of sorafenib has not been systematically examined in HCC patients. Therefore, our study aims to determine whether CYP3A4- and UGT1A9-mediated sorafenib metabolism is differentially affected in HCC patient tumor hepatic microsomes (THLMs) in comparison with those in adjacent normal hepatic microsomes (NHLMs) and commercial hepatic microsomes (CHLMs). CYP3A4 and UGT1A9 protein expression in different tissues were also determined. The results of this study will support valuable information for sorafenib in clinical use.

## Methods

### Ethics Statement

Approvals for tissue collection and in vitro xenobiotic metabolism studies were obtained from the Nanfang Hospital Research Ethics Committee. All the patients with HCC provided their written informed consents to participate in this study.

### Source of Human Liver Tissues

Human liver samples (30 g to 50 g) were obtained from patients with HCC who were undergoing hepatic surgery. Patients didn’t receive any antitumor medication before the surgery. A total of 18 male (aged 39 to 75 years) liver samples were used in the present study. The cases include patients who were admitted from 2011 to 2012 in the Affiliated Nanfang Hospital of Southern Medical University, Guangzhou, China.

### Preparation of Microsomes

Healthy tissues surrounding the primary tumor were isolated and considered as adjacent normal liver tissues. Then, the two parts were separately homogenized in ice-cold Tris-HCl buffer to yield liver homogenate tissue. Microsomal fractions were prepared by differential ultracentrifugation [28]. Part of 18 samples each of THLMs and NHLMs were separately mixed. Male-pooled CHLMs purchased from BD Gentest Corp. (Woburn, MA) were used as a control. Microsome concentration was detected by Bio-Rad protein assay (Bio-Rad, Hercules, CA) as previously described [29].

### Sorafenib Metabolism by CYP3A4 and UGT1A9 in the Different Microsomes

Sorafenib was metabolized with pooled and individual NHLMs, THLMs, or CHLMs in typical phases I and II reaction incubation systems as described previously [30], [31].

### Sorafenib Metabolism by CYP3A4 with the Pooled and Individual Microsomes

A typical phase I incubation system contained potassium phosphate (50 mM, pH 7.4), NADP (1.55 mM), 6-P-G (3.3 mM), MgCl2 (3.3 mM), PDH (0.4 U/mL), pooled HLMs (THLMs, NHLMs or CHLMs, 0.4 mg protein/mL), and sorafenib (0.39∼120 µM) in a total volume of 500 µL. Incubations were carried out for 90 min in a shaking water bath (150 rpm) at 37°C. Termination of the enzyme activity was by the addition of 4 mL ice-cold dichloromethane containing testosterone (used as an internal standard). After centrifugation, the supernatant was drawn into another tube and evaporated to dryness. The residue was dissolved with 50% methanol in water and analyzed by liquid chromatography with tandem mass spectrometry detection (LC-MS/MS). All incubations were performed in triplicate.

In addition, sorafenib metabolism in phase I incubation system with individual sample (n = 18) was tested.

### Sorafenib Metabolism by UGT1A9 with the Pooled and Individual Microsomes

The optimal phase II incubation procedures for measuring UGT activity were as follows: pooled HLMs (THLMs, NHLMs or CHLMs, 0.133 mg/mL), magnesium chloride (0.88 mM), saccharolactone (4.4 mM), alamethicin (0.022 mg/mL), different concentrations of sorafenib (0.078∼40 µM) in a 50 mM potassium phosphate buffer (pH 7.4), and UDPGA (3.5 mM) were mixed and incubated at 37°C for 90 min (the final volume of mixture was 120 µL). The reaction was terminated by the addition of 60 µL acetonitrile containing testosterone as the internal standard. After centrifugation, the supernatant was analyzed by liquid chromatography with tandem mass spectrometry detection (LC-MS/MS). All experiments were conducted in triplicate.

In addition, sorafenib metabolism in phase II incubation system with individual sample (n = 18) was tested.

### Determination Sorafenib and its Metabolites by UPLC-MS/MS

The UPLC conditions: system, Waters Acquity™; column, Acquity UPLC HSS T3 column (100×2.1 mm, 1.8 µm, Waters, Milford, MA, USA); mobile phase A: 0.1% (v/v) formic acid in water; mobile phase B: 100% acetonitrile; gradient, 0 min to 4 min at 50 to 70% B, 4 min to 5 min at 70% to 50% B; flow rate, 0.3 mL/min; column temperature, 50°C; and injection volume, 10 µL. The MS/MS detector used was a quadrupole tandem mass spectrometer (Waters, USA). Samples were analyzed using electrospray ionization in the positive model. The main working parameters were set as follows: capillary voltage, 3 kV; cone voltage, 38 V; collision voltage, 35 V; source temperature, 120°C; desolvation temperature, 400°C; desolvation gas flow, 600 L/Hr; cone gas flow, 50 L/Hr; and collision gas glow, 0.20 mL/min. Data were collected and analyzed by Waters Quantify software (Masslynx 4.1, Waters, USA). Sorafenib was monitored at *m/z* 465.3>252.4. Metabolites including M1, M2, M3, M4, M5 and M7 were monitored at *m/z* 497.0>479.0, *m/z* 481.3>286.3, *m/z* 481.3>268.3, *m/z* 451.0>406.3, *m/z* 467.0>202.0 and *m/z* 641.0>270.2 transitions, respectively. Testosterone was used as an internal standard (IS) and was monitored at *m/z* 289.5>97.4.

### Data Analysis

Data are analyzed using Student’s T-test analysis or One-way analysis of variance (ANOVA) with or without Tukey–Kramer multiple comparisons (post-hoc) tests. Differences are considered statistically significant when *P*<0.05.

Kinetic parameters were estimated by fitting the proper models (Michaelis-Menten, autoactivation (Hill equations), substrate inhibition or biphasic kinetic) to the substrate concentrations and initial rates with a weighting of 1, aided by profiles of the Eadie–Hofstee plots as previously described [32]. When the Eadie–Hofstee plots showed the autoactivation kinetic, the data from these atypical profiles were fit to
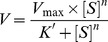
(1)


where *S* is the substrate concentration, *V* is the initial reaction rate, *Vmax* is the maximal enzyme velocity, *K'* is related to the* K*_*m*_ (the substrate concentration at which the reaction rate is half of *Vmax*), and n is the Hill slope.

When Eadie-Hofstee plots revealed substrate inhibition kinetics, the formation rates (*V*) were fit to
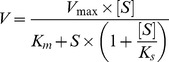
(2)


where *S* is the substrate concentration, *V* is the initial reaction rate, *V_max_* is the maximum enzyme velocity, *K_m_* is the substrate concentration at which the reaction rate is half of *V_max_*, and *K_s_* is the substrate inhibition constant. The goodness of fit was evaluated on the basis of R2 values, AIC (Akaike’s information criteria) and residual plots.

The standard substance of M2 is commercial, in that M2 is the main metabolite of sorafenib mediated by CYP3A4. However, other metabolites couldn’t be absolutely quantified due to none commercial standard substances. Therefore, *V* (Y axis) used only in M2 and other metabolites used peak area ratio instead when we determined the kinetic parameters of sorafenib.

### Immunoquantitation of CYP3A4 and UGT1A9 Protein by Western Blot Analysis

Microsomal proteins were analyzed by SDS-polyacrylamide gel electrophoresis (10% acrylamide gels), and then transferred onto PVDF membranes. Blots were probed with anti-CYP3A4 (Abcam, Cambridge, UK) or anti-UGT1A9 antibody (Santa Cruz Biotechnology, Santa Cruz, CA), followed by HRP-conjugated goat anti-rabbit IgG or HRP-conjugated goat anti-mouse IgG (Santa Cruz Biotechnology, Santa Cruz, CA), respectively. Membranes were developed by chemiluminescence, following the enhanced chemiluminescence protocol (PerkinElmer Inc., Netherland).

## Results

Eight sorafenib metabolites have been identified [2]. Given that M6 and M8 are not detectable by the present analytical method, we determined the formation of sorafenib metabolites M1 to M5 mediated by CYP3A4 and M7 mediated by UGT1A9 in the pooled and individual THLMs, NHLMs, and CHLMs.

**Figure 1:**
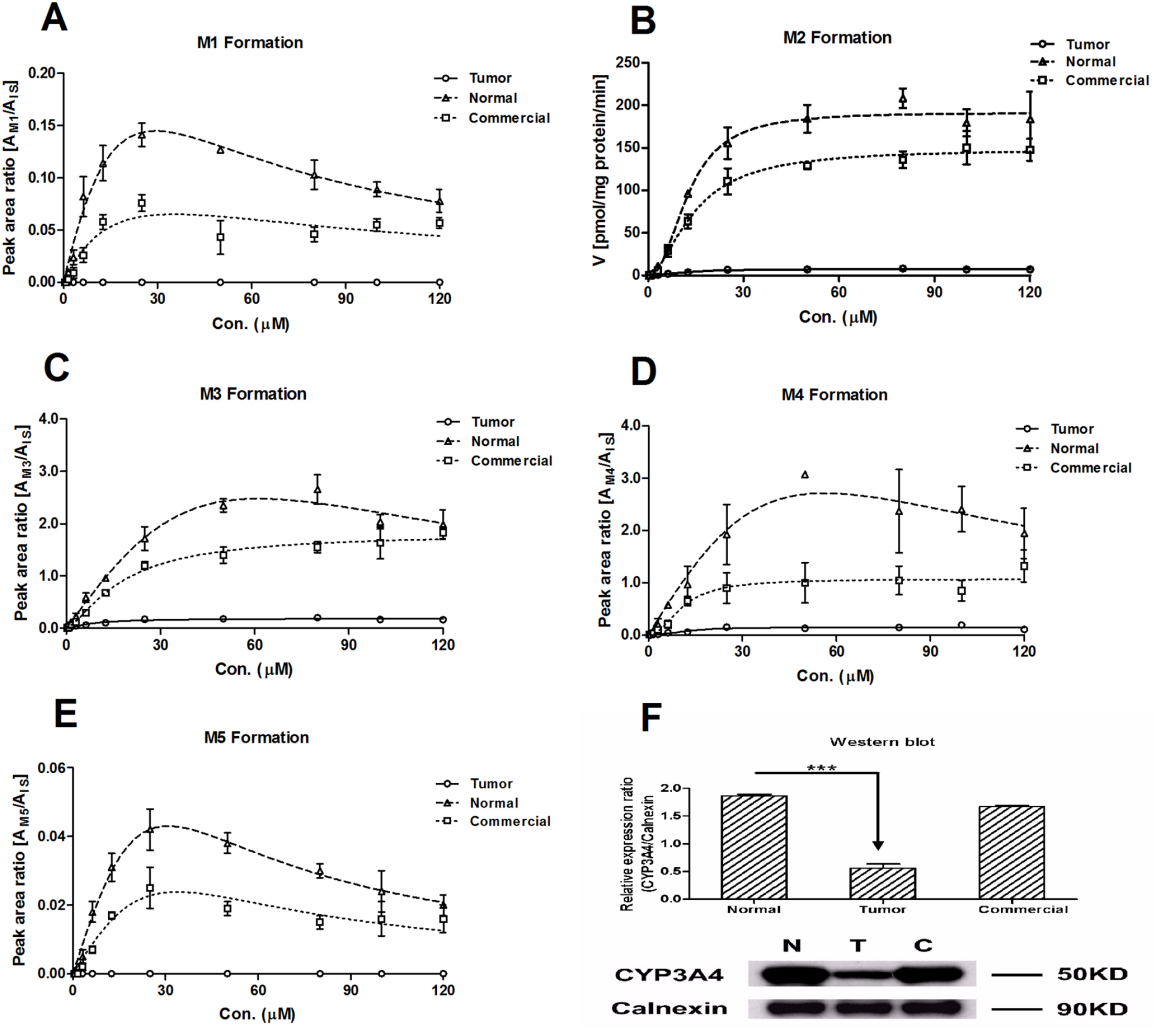
Sorafenib metabolism by CYP3A4 (A–E) and CYP3A4 protein expression (F) in pooled NHLMs, THLMs and CHLMs. The formation rate of M2 expressed as pmol/mg/min (mean ± SD), other metabolites rates (M1, M3, M4 and M5) expressed as peak area ratio (mean ± SD). All the experiments were carried out in triplicate. One-way ANOVA, with or without Tukey–Kramer multiple comparison (post-hoc) tests, was used for data analysis. “*” denotes statistical significance (*P*<0.05); “**” denotes statistical significance (*P*<0.01), “***” denotes statistical significance (*P*<0.001).

Mean rates of CYP3A4 metabolite formation with increasing sorafenib concentration were shown in Figure 1. Sorafenib exhibited autoactivation oxidation kinetics in the pooled THLMs, NHLMs, and CHLMs. The rate of CYP3A4-mediated oxidation of sorafenib was remarkably decreased in the pooled THLMs compared with those in the pooled NHLMs and pooled CHLMs (Figures1A–1E). The intrinsic clearance (*CL*) of sorafenib calculated by M2 formation was 17-fold lower in the pooled THLMs (0.55 versus 9.52 µL/min/mg protein, Table1) as a consequence of a decrease of its *V_max_* of metabolism (7.25±0.19 versus 184.61±2.14 pmol/min/mg protein). No significant differences were observed in the *K_m_* values of sorafenib in the different groups (Table 1), indicating that liver disease did not alter the CYP3A4 enzyme’s affinity for sorafenib. Additionally, we also estimated the *CL* and *K_m_* of sorafenib by other oxidative metabolites using their peak areas ratio. A remarkable decrease (ranging from 7.5- to 15-fold) in the *CL* of pooled THLMs was noted compared with that in the NHLMs (Table 2). CYP3A4 protein expression was down regulated in the THLMs compared with that in the NHLMs and CHLMs (Figure 1F).

**Table 1:** Kinetic parameters of sorafenib calculated by M2 formation in pooled NHLMs, THLMs and CHLMs.

Kinetic Parameters	pooled NHLMs	pooled THLMs	pooled CHLMs
*K'* (µM)	19.08±5.88	13.10±6.85	11.48±4.19
*V_max_* (pmol/min/mg)	184.61±2.14	7.25±0.19	148.90±3.33
*CL* (*V_max_/K'*, µl/min/mg protein)	9.52	0.55	12.97
R^2^	0.999	0.995	0.997
AIC	−48.82	−0.91	−59.31

**Table 2:** Kinetic parameters of sorafenib calculated by other CYP3A4 mediated metabolites in pooled NHLMs, THLMs and CHLMs.

Metabolites	Kinetic Parameters	pooled NHLMs	pooled THLMs	pooled CHLMs
M1	*K_m_* (µM)	-	4.43±2.71	5.94±4.15
	*V_max_* (A_M1_/A_IS_)	-	0.12±0.01	0.06±0.01
	*CL* (*V_max_*/*K_m_*)	-	0.03	0.01
M3	*K_m_* (µM)	9.75±2.53	18.23±6.35	27.66±4.67
	*V_max_* (A_M3_/A_IS_)	0.20±0.01	2.73±0.27	2.17±0.12
	*CL* (*V_max_*/*K_m_*)	0.02	0.15	0.08
M4	*K_m_* (µM)	16.53±9.29	17.39±8.51	17.55±6.63
	*V_max_* (A_M4_/A_IS_)	0.17±0.03	2.90±0.40	1.30±0.14
	*CL* (*V_max_*/*K_m_*)	0.01	0.17	0.07
M5	*K_m_* (µM)	-	5.03±3.52	7.11±4.38
	*V_max_* (A_M5_/A_IS_)	-	0.033±0.005	0.020±0.002
	*CL* (*V_max_*/*K_m_*)	-	0.007	0.003

- not detectable.

**Figure 2:**
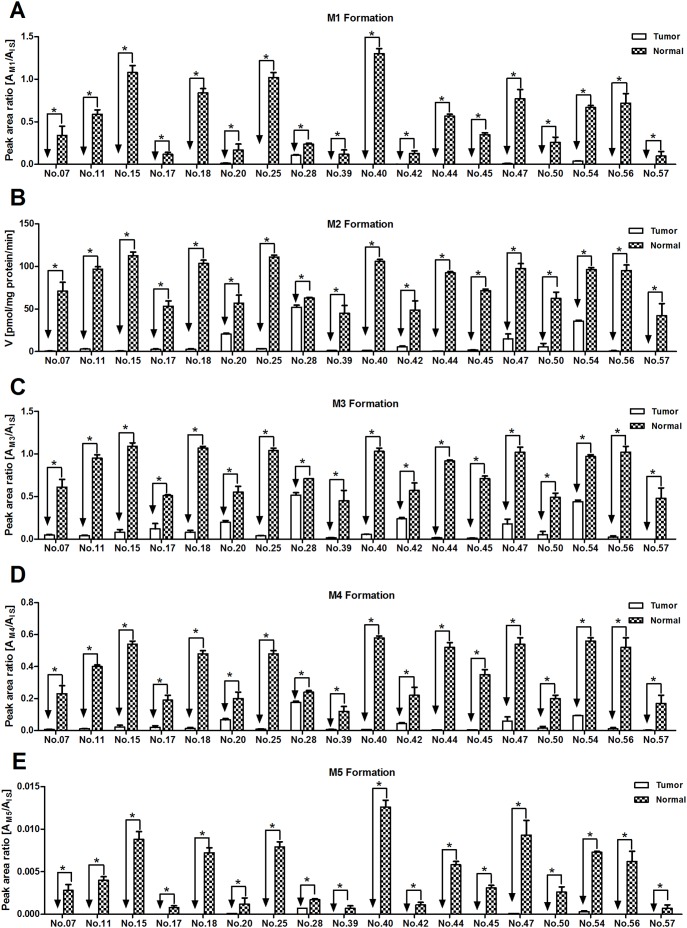
Inter-individual variability of sorafenib metabolism by CYP3A4 with 18 human liver microsomes samples (THLMs and NHLMs). Sorafenib (15 µM) was incubated with the microsomes obtained from different patients for 90 min at 37°C (the micorsome concentration was 0.4 mg/ml). The formation rate of M2 expressed as pmol/mg/min (mean ± SD), other metabolites rates (M1, M3, M4 and M5) expressed as peak area ratio (mean ± SD). All the experiments were performed in triplicate. Student’s T-test analysis was used for data analysis. “*” denotes statistical significance (*P*<0.05).

Individual metabolism mediated by CYP3A4 was depicted in Figure 2. The results showed that the amounts of all the CYP3A4 metabolites of sorafenib in each patient’s THLMs sample were significantly lower (ranging from 7 to 67-fold) than those in corresponding NHLMs sample (Figure 2). More specifically, the average amount of M2 formed in NHLMs was 79.20±24.51 pmol/min/mg protein (varying from 42.32 to 112.66 pmol/min/mg protein), while that in THLMs was 8.46±14.06 pmol/mg protein/min (ranging from 0.076 to 52.08 pmol/min/mg protein).

**Figure 3:**
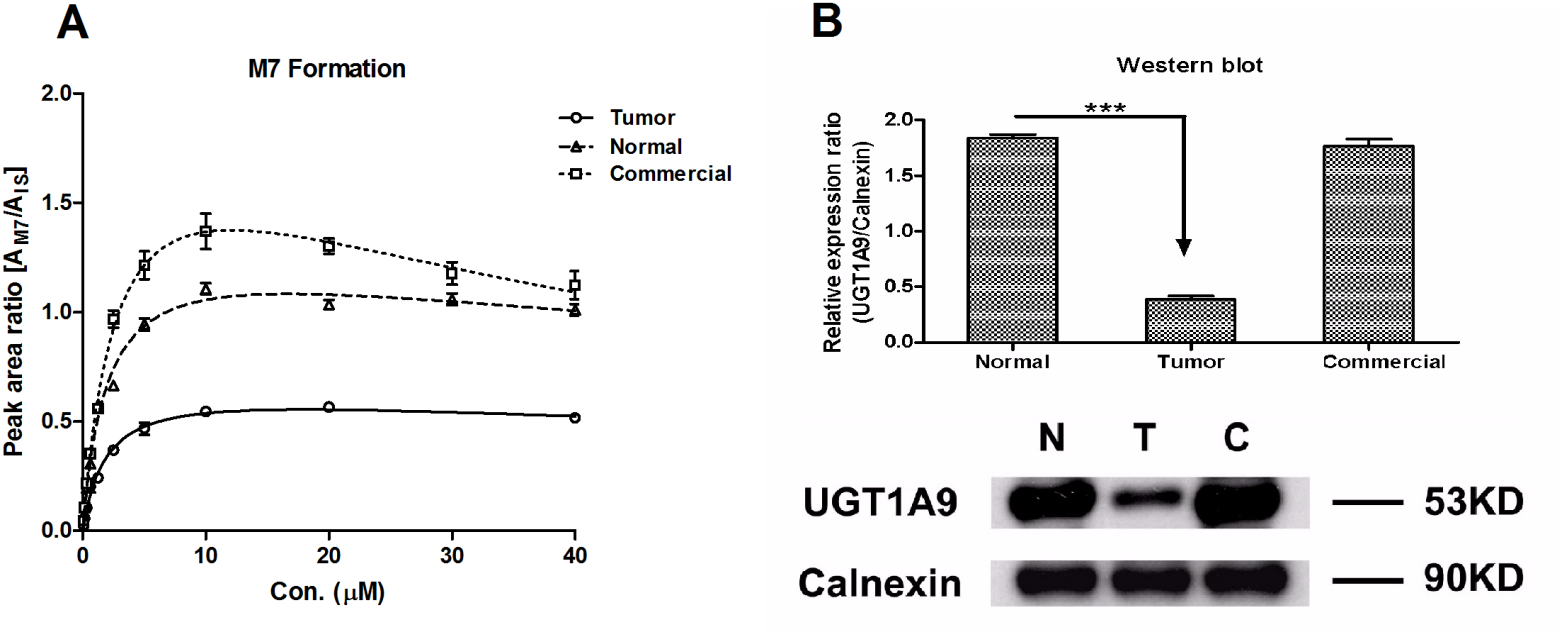
Sorafenib metabolism by UGT1A9 (A) and UGT1A9 protein expression (B) in pooled NHLMs, THLMs and CHLMs. The formation rate of M7 expressed as peak area ratio (mean ± SD). All the experiments were carried out in triplicate. One-way ANOVA, with or without Tukey–Kramer multiple comparison (post-hoc) tests, was used for data analysis. “*” denotes statistical significance (*P*<0.05); “**” denotes statistical significance (*P*<0.01), “***” denotes statistical significance (*P*<0.001).

Mean rates of UGT1A9 metabolite formation with increasing sorafenib concentration were shown in Figure 3. Sorafenib showed substrate inhibition glucuronidation kinetics in the pooled THLMs, NHLMs, and CHLMs. In sorafenib glucuronidation, M7 in the pooled THLMs demonstrated an expected remarkable decrease in contrast to that in the pooled NHLMs and CHLMs (Figure 3A). As expected, the *V_max_* value of sorafenib calculated by M7 formation was significantly decreased in the pooled THLMs. Afterwards, the *CL* in the pooled THLMs was 1.9-fold lower than that in the pooled NHLMs (Table 3). There was no change in the *K_m_* values of sorafenib for the two groups, suggesting that liver disease did not change the UGT1A9 enzyme’s affinity for sorafenib (Table 3). Correspondingly, the protein level of UGT1A9 in THLMs revealed a considerable decrease, comparing to NHLMs and CHLMs (Figure 3B).

**Table 3:** Kinetic parameters of sorafenib glucuronidation calculated by M7 formation in pooled NHLMs, THLMs and CHLMs.

Kinetic Parameters	pooled NHLMs	pooled THLMs	pooled CHLMs
*K_m_* (µM)	1.97±0.31	1.89±0.26	2.89±0.29
*V_max_* (A_M7_/A_IS_)	1.34±0.09	0.67±0.037	2.03±0.10
*K*_s_ (µM)	142.9±55.7	172.3±68.6	50.6±6.9
*CL* (*V_max_/K_m_)*	0.68	0.35	0.70
R^2^	0.991	0.994	0.997
AIC	−40.42	−53.78	−48.21

**Figure 4:**
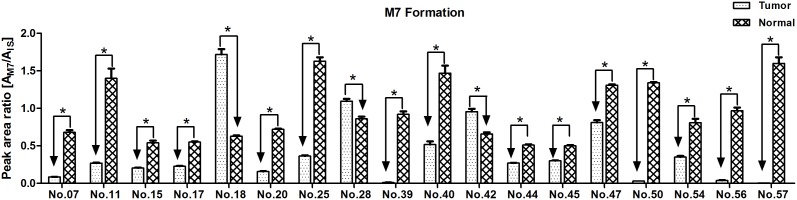
Inter-individual variability of sorafenib metabolism by UGT1A9 with 18 human liver microsomes samples (THLMs and NHLMs). Sorafenib (2.5 µM) was incubated with microsomes (0.133 mg/ml) obtained from different patients for 90 min at 37°C. The formation rate of M7 expressed as peak area ratio (mean ± SD). All the experiments were performed in triplicate. Student’s T-test analysis was used for data analysis. “*” denotes statistical significance (*P*<0.05).

Similarly, we also investigated sorafenib metabolism by UGT1A9 with individual THLMs and NHLMs (n = 18). The amount of M7 in each patient THLMs sample was significantly lower than that in corresponding NHLMs sample (Figure 4), except the samples of three patients (No. 18, 28 and 42). Detailedly, the average formation of M7 (Area_M7_/Area_IS_) in THLMs was 2-fold less than that in NHLMs (0.41±0.45 versus 0.95±0.39).

## Discussion

Previous publications have demonstrated that the metabolism of many drugs has considerably altered in the patients with liver disease [17], [26], [27]. Sorafenib is the first line treatment drug for the advanced HCC. Little attention has been done on its metabolism characteristic in HCC patients. Our present study indicated the mean rates of oxidation mediated by CYP3A4 and glucuronidation mediated by UGT1A9 of sorafenib were significantly decreased in the pooled THLMs, compared with those in NHLMs and CHLMs. Noteworthy, maximal velocity (*V_max_*) of sorafenib oxidation and glucuronidation were approximately 25-fold and 2-fold decreased in the pooled THLMs, respectively, with unchanged *K_m_* values. The disease affected the apparent *V_max_*, but not *K_m_*, suggesting that the level of enzyme expression was impaired. Herein, we determined the level of expression of CYP3A4 and UGT1A9 by western blotting. As expected, the level of CYP3A4 and UGT1A9 expression were both notably decreased in the pooled THLMs.

Individual metabolism of sorafenib was also tested in our present study. Among the 18 patients’ samples, the CYP3A4-mediated oxidation of sorafenib was all decreased in THLMs. Nevertheless, the reduction of UGT1A9-mediated glucuronidation (M7) in THLMs was observed in 15 out of 18 patients’ samples. In the remaining three samples (No. 18, No. 28 and No. 42), the glucuronidation in THLMs was greater than that in NHLMs. We believe we determined accurately differences in glucuronidation activities, even though we could not quantify the formation rate of M7 using standards, since the activity measurements were done under linear assay conditions and using a substrate concentration equal to the *K_m_* determined under the experimental conditions reported in this study. In addition, we used commercial pooled human microsomes (CHLMs) as a control. In CHLMs, the *K_m_* value was 11.48±4.19 µM for oxidation and 2.89±0.29 µM for glucuronidation, which was equal to that reported in the previous publication (12.1±0.71 µM and 3.6±0.22 µM, respectively) [33]. Furthermore, we have measured the UGT1A9 protein content by UPLC-MS/MS. And the results shown that UGT1A9 protein expression in THLMs of these three patients was greater than that in corresponding NHLMs (data not shown, due to these data were using in another related manuscript), which was accordance with the observed UGT1A9 activity.

As is well known, there are large differences in UGT expression in different individuals, and the variability in expression is a major determinant of glucuronidation capacity. Many factors are considered to affect the variability of UGT expression, such as diet, smoking behavior, co-medication and diseases. However, the liver-enriched transcription factors (LETFs) have a major role in regulation UGT expression in the liver [34]. UGT1A9, 6.1% of the total expression of UGT enzymes with hepatic tissue, is the second abundant isoform among UGT1A subfamily in the liver [35], [36]. HNF1α, HNF4α, and Cdx2 have been shown to regulate the hepatic transcription of the UGT1A9 gene. Transcription factor levels are known to vary between individuals. For example, the level of HNF1α mRNA in human liver varies up to 10-fold [37]. In addition, stimuli that alter the expression or activity of LETFs may also change the expression of UGT1A9. Furthermore, Polymorphisms in the genes coding for LETFs or their co-factors, or in their cognate binding sites, may affect UGT1A9 expression [38].

It was reported that the single-nucleotide polymorphisms in positions T-440C/C-331T, C-665T and T-1887G in the promoter region, and M^33^T of UGT1A9*3 alleles significantly enhanced mycophenolic acid glucuronidation in vitro [39]–[41]. Besides, UGT1A9 intronic 1399 C>T polymorphism enhanced SN-38 glucuronidation in Asian cancer patients [42]. Taken together, the abnormal phenomenon of these three patients might have a much more related with polymorphism of UGT1A9.

Liver disease may have intricate roles in drug pharmacokinetics, biotransformation and clearance. Sometimes alterations increase the bioavailability of drug, causing normal drug doses to have toxic effects. Sorafenib-related HFSR and other side effects are associated with increasing cumulative sorafenib dose [4]–[6]. Sorafenib metabolism is significantly altered in the liver tumor tissue of HCC patient. The decreased metabolism of sorafenib might have a potential to increase the plasma concentration of sorafenib and its metabolites, resulting in sorafenib-induced HFSR and other side effects. Although the metabolic activity and expression level of CYP3A4 and UGT1A9 were low exclusively in THLMs but not in NHLMs in our patients, the plasma concentrations of sorafenib and its metabolites probably depend on the volume of HCC tumor tissue and/or the surrounding cirrhotic tissue. Further studies have to be performed to clarify whether the specific metabolic features of sorafenib in HCC tumor tissue contribute to increase their plasma concentrations and to cause HFSR.

In conclusion, we determined the metabolism features of sorafenib in HCC patients. Sorefenib metabolism is significantly altered in the liver tumor tissue of HCC patient, due to a remarkable decrease of the expression level of CYP3A4 and UGT1A9. The mechanisms involved in the downregulation of these enzymes and the relationship between altered sorafenib metabolism in HCC tumor tissue and the clinical beneficial and adverse effects of sorafenib in HCC patients should be examined in future studies.

## References

[1] Villeneuve JP, Pichette V (2004) Cytochrome P450 and liver diseases. Curr Drug Metab 5: 273–282. 15180496 10.2174/1389200043335531

[2] Keating GM, Santoro A (2009) Sorafenib: a review of its use in advanced hepatocellular carcinoma. Drugs 69: 223–240. 19228077 10.2165/00003495-200969020-00006

[3] Cheng AL, Kang YK, Chen Z, Tsao CJ, Qin S, et al (2009) Efficacy and safety of sorafenib in patients in the Asia-Pacific region with advanced hepatocellular carcinoma: a phase III randomised, double-blind, placebo-controlled trial. Lancet Oncol 10: 25–34. 19095497 10.1016/S1470-2045(08)70285-7

[4] Chu D, Lacouture ME, Fillos T, Wu S (2008) Risk of hand-foot skin reaction with sorafenib: a systematic review and meta-analysis. Acta Oncol 47: 176–186. 18210295 10.1080/02841860701765675

[5] Chung NM, Gutierrez M, Turner ML (2006) Leukocytoclastic vasculitis masquerading as hand-foot syndrome in a patient treated with sorafenib. Arch Dermatol 142: 1510–1511. 17116852 10.1001/archderm.142.11.1510

[6] Wolber C, Udvardi A, Tatzreiter G, Schneeberger A, Volc-Platzer B (2009) Perforating folliculitis, angioedema, hand-foot syndrome–multiple cutaneous side effects in a patient treated with sorafenib. J Dtsch Dermatol Ges 7: 449–452. 19178612 10.1111/j.1610-0387.2009.07017.x

[7] Wang Z, Zhou J, Fan J, Qiu SJ, Yu Y, et al (2008) Effect of rapamycin alone and in combination with sorafenib in an orthotopic model of human hepatocellular carcinoma. Clin Cancer Res 14: 5124–5130. 18698030 10.1158/1078-0432.CCR-07-4774

[8] Kolwankar D, Vuppalanchi R, Ethell B, Jones DR, Wrighton SA, et al (2007) Association between nonalcoholic hepatic steatosis and hepatic cytochrome P-450 3A activity. Clin Gastroenterol Hepatol 5: 388–393. 17368239 10.1016/j.cgh.2006.12.021

[9] Clark JW, Eder JP, Ryan D, Lathia C, Lenz HJ (2005) Safety and pharmacokinetics of the dual action Raf kinase and vascular endothelial growth factor receptor inhibitor, BAY 43-9006, in patients with advanced, refractory solid tumors. Clin Cancer Res 11: 5472–5480. 16061863 10.1158/1078-0432.CCR-04-2658

[10] Peer CJ, Sissung TM, Kim A, Jain L, Woo S, et al (2012) Sorafenib is an inhibitor of UGT1A1 but is metabolized by UGT1A9: implications of genetic variants on pharmacokinetics and hyperbilirubinemia. Clin Cancer Res 18: 2099–2107. 22307138 10.1158/1078-0432.CCR-11-2484 PMC6432766

[11] Gomo C, Coriat R, Faivre L, Mir O, Ropert S, et al (2010) Pharmacokinetic interaction involving sorafenib and the calcium-channel blocker felodipine in a patient with hepatocellular carcinoma. Invest New Drugs 29: 1511–1514. 20706860 10.1007/s10637-010-9514-3

[12] Noda S, Shioya M, Hira D, Fujiyama Y, Morita SY, et al (2013) Pharmacokinetic interaction between sorafenib and prednisolone in a patient with hepatocellular carcinoma. Cancer Chemother Pharmacol 72: 269–272. 23673446 10.1007/s00280-013-2187-9

[13] Gemignani F, Landi S, Szeszenia-Dabrowska N, Zaridze D, Lissowska J, et al (2007) Development of lung cancer before the age of 50: the role of xenobiotic metabolizing genes. Carcinogenesis 28: 1287–1293. 17259654 10.1093/carcin/bgm021

[14] Vidjaya Letchoumy P, Chandra Mohan KV, Stegeman JJ, Gelboin HV, Hara Y, et al (2008) Pretreatment with black tea polyphenols modulates xenobiotic-metabolizing enzymes in an experimental oral carcinogenesis model. Oncol Res 17: 75–85. 18543609 10.3727/096504008784523649 PMC3974168

[15] Yang LQ, Li SJ, Cao YF, Man XB, Yu WF, et al (2003) Different alterations of cytochrome P450 3A4 isoform and its gene expression in livers of patients with chronic liver diseases. World J Gastroenterol 9: 359–363. 12532467 10.3748/wjg.v9.i2.359 PMC4611347

[16] Niu Y, Wu Z, Shen Q, Song J, Luo Q, et al.. (2013) Hepatitis B virus X protein co-activates pregnane X receptor to induce the cytochrome P450 3A4 enzyme, a potential implication in hepatocarcinogenesis. Dig Liver Dis. 10.1016/j.dld.2013.06.004 23891548

[17] Furlan V, Demirdjian S, Bourdon O, Magdalou J, Taburet AM (1999) Glucuronidation of drugs by hepatic microsomes derived from healthy and cirrhotic human livers. J Pharmacol Exp Ther 289: 1169–1175. 10215701

[18] Chalasani N, Gorski JC, Patel NH, Hall SD, Galinsky RE (2001) Hepatic and intestinal cytochrome P450 3A activity in cirrhosis: effects of transjugular intrahepatic portosystemic shunts. Hepatology 34: 1103–1108. 11731998 10.1053/jhep.2001.29306

[19] Lown K, Kolars J, Turgeon K, Merion R, Wrighton SA, et al (1992) The erythromycin breath test selectively measures P450IIIA in patients with severe liver disease. Clin Pharmacol Ther 51: 229–238. 1544283 10.1038/clpt.1992.17

[20] Huet PM, Villeneuve JP (1983) Determinants of drug disposition in patients with cirrhosis. Hepatology 3: 913–918. 6629320 10.1002/hep.1840030604

[21] Chalon SA, Desager JP, Desante KA, Frye RF, Witcher J, et al (2003) Effect of hepatic impairment on the pharmacokinetics of atomoxetine and its metabolites. Clin Pharmacol Ther 73: 178–191. 12621383 10.1067/mcp.2003.25

[22] Arns PA, Adedoyin A, DiBisceglie AM, Waggoner JG, Hoofnagle JH, et al (1997) Mephenytoin disposition and serum bile acids as indices of hepatic function in chronic viral hepatitis. Clin Pharmacol Ther 62: 527–537. 9390109 10.1016/S0009-9236(97)90048-5

[23] Giannini E, Fasoli A, Chiarbonello B, Malfatti F, Romagnoli P, et al (2002) 13C-aminopyrine breath test to evaluate severity of disease in patients with chronic hepatitis C virus infection. Aliment Pharmacol Ther 16: 717–725. 11929389 10.1046/j.1365-2036.2002.01200.x

[24] Pique JM, Feu F, de Prada G, Rohss K, Hasselgren G (2002) Pharmacokinetics of omeprazole given by continuous intravenous infusion to patients with varying degrees of hepatic dysfunction. Clin Pharmacokinet 41: 999–1004. 12222996 10.2165/00003088-200241120-00004

[25] Villeneuve JP, Infante-Rivard C, Ampelas M, Pomier-Layrargues G, Huet PM, et al (1986) Prognostic value of the aminopyrine breath test in cirrhotic patients. Hepatology 6: 928–931. 3758945 10.1002/hep.1840060520

[26] Pasanen M, Rannala Z, Tooming A, Sotaniemi EA, Pelkonen O, et al (1997) Hepatitis A impairs the function of human hepatic CYP2A6 in vivo. Toxicology 123: 177–184. 9355936 10.1016/s0300-483x(97)00119-4

[27] Witassek F, Bircher J, Huguenin P, Preisig R (1983) Abnormal glucuronidation of zomepirac in patients with cirrhosis of the liver. Hepatology 3: 415–422. 6840687 10.1002/hep.1840030322

[28] Chen J, Halls SC, Alfaro JF, Zhou Z, Hu M (2004) Potential beneficial metabolic interactions between tamoxifen and isoflavones via cytochrome P450-mediated pathways in female rat liver microsomes. Pharm Res 21: 2095–2104. 15587933 10.1023/b:pham.0000048202.92930.61

[29] Chen J, Lin H, Hu M (2003) Metabolism of flavonoids via enteric recycling: role of intestinal disposition. J Pharmacol Exp Ther 304: 1228–1235. 12604700 10.1124/jpet.102.046409

[30] Ye L, Yang XS, Lu LL, Chen WY, Zeng S, et al (2013) Monoester-Diterpene Aconitum Alkaloid Metabolism in Human Liver Microsomes: Predominant Role of CYP3A4 and CYP3A5. Evid Based Complement Alternat Med 2013: 941093. 23864901 10.1155/2013/941093 PMC3705941

[31] Tang L, Singh R, Liu Z, Hu M (2009) Structure and concentration changes affect characterization of UGT isoform-specific metabolism of isoflavones. Mol Pharm 6: 1466–1482. 19545173 10.1021/mp8002557 PMC2941769

[32] Houston JB, Kenworthy KE (2000) In vitro-in vivo scaling of CYP kinetic data not consistent with the classical Michaelis-Menten model. Drug Metab Dispos 28: 246–254. 10681367

[33] Zimmerman EI, Roberts JL, Li L, Finkelstein D, Gibson A, et al (2012) Ontogeny and sorafenib metabolism. Clin Cancer Res 18: 5788–5795. 22927483 10.1158/1078-0432.CCR-12-1967 PMC3490489

[34] Gardner-Stephen DA, Mackenzie PI (2008) Liver-enriched transcription factors and their role in regulating UDP glucuronosyltransferase gene expression. Current drug metabolism 9: 439–452. 18537579 10.2174/138920008784746409

[35] Rowland A, Miners JO, Mackenzie PI (2013) The UDP-glucuronosyltransferases: their role in drug metabolism and detoxification. The international journal of biochemistry & cell biology 45: 1121–1132. 23500526 10.1016/j.biocel.2013.02.019

[36] Wu B, Kulkarni K, Basu S, Zhang S, Hu M (2011) First-pass metabolism via UDP-glucuronosyltransferase: a barrier to oral bioavailability of phenolics. Journal of pharmaceutical sciences 100: 3655–3681. 21484808 10.1002/jps.22568 PMC3409645

[37] Toide K, Takahashi Y, Yamazaki H, Terauchi Y, Fujii T, et al (2002) Hepatocyte nuclear factor-1α is a causal factor responsible for interindividual differences in the expression of UDP-glucuronosyltransferase 2B7 mRNA in human livers. Drug metabolism and disposition 30: 613–615. 12019184 10.1124/dmd.30.6.613

[38] Girard H, Bernard O, Fortier L-C, Villeneuve L, Hao Q, et al (2004) Identification of common polymorphisms in the promoter of the UGT1A9 gene: evidence that UGT1A9 protein and activity levels are strongly genetically controlled in the liver. Pharmacogenetics and Genomics 14: 501–515. 10.1097/01.fpc.0000114754.08559.27 15284532

[39] Villeneuve L, Girard H, Fortier LC, Gagne JF, Guillemette C (2003) Novel functional polymorphisms in the UGT1A7 and UGT1A9 glucuronidating enzymes in Caucasian and African-American subjects and their impact on the metabolism of 7-ethyl-10-hydroxycamptothecin and flavopiridol anticancer drugs. The Journal of pharmacology and experimental therapeutics 307: 117–128. 12944498 10.1124/jpet.103.054072

[40] Fukuda T, Goebel J, Cox S, Maseck D, Zhang K, et al (2012) UGT1A9, UGT2B7, and MRP2 genotypes can predict mycophenolic acid pharmacokinetic variability in pediatric kidney transplant recipients. Therapeutic drug monitoring 34: 671–679. 23131697 10.1097/FTD.0b013e3182708f84 PMC3498586

[41] Bernard O, Guillemette C (2004) The main role of UGT1A9 in the hepatic metabolism of mycophenolic acid and the effects of naturally occurring variants. Drug metabolism and disposition: the biological fate of chemicals 32: 775–778. 15258099 10.1124/dmd.32.8.775

[42] Sandanaraj E, Jada SR, Shu X, Lim R, Lee SC, et al (2008) Influence of UGT1A9 intronic I399C>T polymorphism on SN-38 glucuronidation in Asian cancer patients. The pharmacogenomics journal 8: 174–185. 17700594 10.1038/sj.tpj.6500473

